# Microbial Ecology and Global Health

**DOI:** 10.1155/2011/564701

**Published:** 2011-04-28

**Authors:** Max Teplitski, Jorge H. Leitão, Shlomo Sela

**Affiliations:** ^1^Soil and Water Sciences Department, Genetics Institute, University of Florida-IFAS, Gainesville, FL 32611, USA; ^2^Instituto de Biotecnologia e Biogenharia, Centro de Engenharia Biológica e Química, Instituto Superior Técnico, Portugal; ^3^Microbial Food Safety Research Laboratory, Department of Food Quality and Safety, Agricultural Research Organization (ARO), The Volcani Center, Bet Dagan, Israel

This special issue of the International Journal of Microbiology focuses on global health. More specifically, our aim is to establish whether there is a role for microbial ecology in global health. “Global health” is often defined as the “health of populations” a problem that transcends geographic boundaries and exists at the intersection of demography, economics, epidemiology, political economy, and sociology. Conspicuously absent from this definition is microbial ecology. Is there, after all, a role for microorganisms in health of populations or is “global health” defined strictly by its human dimensions? We argue that the analyses of the factors that led to the emergence and reemergence of pathogens, expansion of vector-transmitted diseases into the new areas of the planet, and recent zoonotic outbreaks highlight an important role for microbial ecology in the health of human populations. Original research and review articles within this special issue contribute to the scientific scaffolding, upon which the risk assessment and management framework is to be built.

Nowhere on this planet are issues of microbial and human ecology more important than in large urban slums. There, humans and microbes do not only intersect, they collide, and the consequences of this collision are often catastrophic. A review by Abraham traces pathogenic, often drug-resistant microbes from inadequate sanitation facilities in megapolises through rivers toward downstream consumers of tainted waters. The review addresses persistence of pathogens in rivers running through the world's major cities, interactions of waterborne pathogens with highly diverse aquatic microbiota, their survival in favorable environmental niches, in biofilms, and in association with amoeba. The author then comments on the utility of indicator organisms in assessing quality of waters in megacities and offers his vision of the pressing research needs for science-based solutions for the water quality and sanitation crisis in the world's urban population centers.

A critical review by N. J. Rowan, builds a case for the need to adapt risk assessment and management framework to accommodate viable but nonculturable (VBNC) pathogens. N. J. Rowan*'s* review contains a thoughtful analysis of the methods for the detection and quantification of viable but non-culturable (VBNC) pathogens and *Cryptosporidium *oocysts, estimating virulence potential of the pathogens in the VBNC state and developing risk assessment models that take into consideration viable but non-culturable as well as drug-resistant pathogens. N. J. Rowan also offers a critical analysis of the efficacy of new water sanitation technologies and the potential impacts of their byproducts on water quality. 

Issues associated with the usefulness of indicator organisms arise again in the review by Bienfang et al. The first part of the review by P. K. Bienfang et al. contains a thorough survey of marine dinoflagellates and cyanobacteria that produce toxic secondary metabolites, and comments on the hypotheses which link production of toxins with various environmental factors. The second part of their review is dedicated to infectious marine microorganisms. This discussion is framed by the five dilemmas associated with the use of indicator organisms to estimate the presence of infectious (introduced and autochthonous) pathogens in seawater. 

Autochthonous opportunistic pathogens take the center stage in the review by S. A. Sousa et al. and the environmental survey of T. Broman et al. Over a 3-year period, T. Broman et al. tested nearly six hundred water and sediment samples and almost one hundred wild rodents for the presence of endemic strains of *Francisella tularensis*. This study demonstrates that culturable *F. tularensis* is widely distributed in the environment. It tests the utility of certain genetic markers for the discrimination between potentially pathogenic and nonpathogenic environmental isolates of *Francisella*. S. A. Sousa et al. provide a thorough review of the epidemiological, comparative, and, functional genomics studies carried out with environmental and pathogenic members of the *Burkholderia cepacia *complex. The latter part of the review by S. A. Sousa et al. contains a critical analysis of the methods that are currently available to the researchers for the identification of virulence determinants in isolates of *B. cepacia* and for the determination of mechanisms which these pathogens use to proliferate in the different environments and infect its vertebrate and invertebrate hosts. 

One of the most disturbing realizations to which scientists and lay people came within the last decade is that human pathogens, like enterovirulent *E. coli* and *Salmonella* can persist in soils and in association with plants for extended periods of time. Consumption of fruits and vegetables, which harbor these human pathogens has already resulted in dozens of large international outbreaks of gastroenteritis. Not surprisingly, therefore, there has been an explosion in research aimed at understanding behavior of *Salmonella* and enterovirulent *E. coli* outside of their human or animal hosts. Research articles by A. D. Duffit et al. and A. M. Ibekwe et al. further contribute to our understanding of the ecology of enterohemorrhagic *E. coli *O157:H7 in soils and water by defining gene expression associated with the persistence in these environments, in the presence (or absence) of fumigants or native soil microbiota. A. A. Mafu et al. correlate attachment of *Salmonella *and other human pathogens to abiotic surfaces with culture conditions and surface properties, thus furthering our understanding of persistence of human pathogens outside of their hosts.

A review by S. Schjørring and K. A. Krogfelt and a research article by S. S. Hussey et al. address the impact of antibiotics on human gut-associated microbiota. The study by S.S. Hussey et al. demonstrates that a short-term parenteral antibiotic administration to human neonates can affect the composition and diversity of the gut associated microbiota. The focus of S. Schjørring and K. A. Krogfelt's review on antibiotic-resistance gene transfer between native gut microbiota and enteric pathogens echoes the reviews by R. Abraham and N. J. Rowan, which appear earlier in the issue, although ecological scales of these three reviews are decidedly different. S. Schjørring and K. A. Krogfelt also tackle the issue of antibiotic resistance in the food production environment, further contributing to our understanding of the ecology of human enteric pathogens outside of their animal hosts. 

Microorganisms are abundant on this planet. Microbial processes like photosynthesis and nitrogen fixation continue to shape this planet. The Plague, Irish Potato Famine, cholera—no textbook is complete without these events that shaped human history. Our history textbooks, however, never mention by name the microorganisms that reshaped continents and almost halted civilizations. In trying to understand these events, we exile their culprits into anonymous infamy and thus diminish the role of ecology of common soil and water microorganisms in the health of human populations. Each year throughout the past century, humans were more and more assertive in defining our global environment. We rarely, however, reflect on the question of how global climate change, population dynamics, methods of food production, crowding in megacities, changes in life styles, and dietary habits affect the ecology of planet's microbiota, the microbiota that shaped this planet, affected global health and often defined the course of human civilizations.

*Max Teplitski* *Max Teplitski* *Jorge H. Leitão* *Jorge H. Leitão* *Shlomo Sela* *Shlomo Sela*

